# The Sulfamate Small Molecule CAIX Inhibitor S4 Modulates Doxorubicin Efficacy

**DOI:** 10.1371/journal.pone.0161040

**Published:** 2016-08-11

**Authors:** Simon J. A. van Kuijk, Roben G. Gieling, Raymon Niemans, Natasja G. Lieuwes, Rianne Biemans, Brian A. Telfer, Guido R. M. M. Haenen, Ala Yaromina, Philippe Lambin, Ludwig J. Dubois, Kaye J. Williams

**Affiliations:** 1 Department of Radiation Oncology (MAASTRO Lab), GROW–School for Oncology and Developmental Biology, Maastricht University Medical Centre, Maastricht, the Netherlands; 2 Hypoxia and Therapeutics Group, Manchester Pharmacy School, University of Manchester, Manchester, United Kingdom; 3 Department of Toxicology, NUTRIM–School for Nutrition, Toxicology, and Metabolism, Maastricht University Medical Center, Maastricht, the Netherlands; University of South Alabama Mitchell Cancer Institute, UNITED STATES

## Abstract

Carbonic anhydrase IX (CAIX) is a tumor-specific protein that is upregulated during hypoxic conditions where it is involved in maintaining the pH balance. CAIX causes extracellular acidification, thereby limiting the uptake of weak basic chemotherapeutic agents, such as doxorubicin, and decreasing its efficacy. The aim of this study was to determine if doxorubicin efficacy can be increased when combined with the selective sulfamate CAIX inhibitor S4. The effect of S4 on doxorubicin efficacy was tested *in vitro* using cell viability assays with MDA-MB-231, FaDu, HT29 –CAIX high and HT29 –CAIX low cell lines. In addition, the efficacy of this combination therapy was investigated in tumor xenografts of the same cell lines. The addition of S4 *in vitro* increased the efficacy of doxorubicin in the MDA-MB-231 during hypoxic exposure (IC50 is 0.25 versus 0.14 µM, p = 0.0003). Similar results were observed for HT29—CAIX high with S4 during normoxia (IC50 is 0.20 versus 0.08 µM, p<0.0001) and in the HT29 –CAIX low cells (IC50 is 0.09 µM, p<0.0001). *In vivo* doxorubicin treatment was only effective in the MDA-MB-231 xenografts, but the efficacy of doxorubicin was decreased when combined with S4. In conclusion, the efficacy of doxorubicin treatment can be increased when combined with the selective sulfamate CAIX inhibitor S4 *in vitro* in certain cell lines. Nevertheless, in xenografts S4 did not enhance doxorubicin efficacy in the FaDu and HT29 tumor models and decreased doxorubicin efficacy in the MDA-MB-231 tumor model. These results stress the importance of better understanding the role of CAIX inhibitors in intratumoral pH regulation before combining them with standard treatment modalities, such as doxorubicin.

## Introduction

The immature and inadequate vasculature of solid tumors prevents oxygen and nutrients supply to certain areas in those tumors. This hypoxic microenvironment selects for a more aggressive tumor phenotype and promotes invasion, migration, and thereby metastases formation [1, 2]. These hypoxic tumor cells are known to be more resistant to standard treatment modalities (e.g. radio- and chemotherapy), highlighting the importance of developing new, or increasing the efficacy of already available, therapies to specifically target hypoxic tumor cells [2–5].

To meet with the high energy demand, tumor cells switch their energy metabolism to glycolysis in hypoxic but also in well-oxygenated areas in the tumors, i.e. the Warburg effect. The increase in glycolytic energy production leads to the production of high concentrations of acids (e.g. lactate and carbon dioxide) [6]. To aid in maintaining cellular pH homeostasis, carbonic anhydrase IX (CAIX) expression becomes transcriptionally upregulated through stabilization of hypoxia-inducible factor 1 (HIF1-α), although alternative hypoxia responses also play a role [7]. CAIX catalyzes the conversion of water and the cellular produced carbon dioxide to bicarbonate and protons. The protons contribute to the hostile acidic extracellular environment, whereas the bicarbonate is transported back intracellularly to aid in maintaining a slightly alkaline pH [8, 9]. The important role that CAIX plays in maintaining the cellular pH balance combined with its predominant expression on hypoxic tumor cells make it an attractive target for treatment [2, 4, 5, 10]. The importance of CAIX on tumors is strengthened further by the significant association between high tumoral CAIX expression and a worse prognosis in patients with many different cancer types [11]. CAIX expression might therefore also be a valuable imaging tool for future clinical practice [12–14]. Previously, several CAIX inhibitors have been shown to be effective in reducing primary tumor growth *in vivo*, either as a single treatment or in combination with radiotherapy [15–17].

CAIX inhibition is also able to increase the efficacy of certain chemotherapeutics, such as the anthracycline antitumor antibiotic doxorubicin [18, 19]. This chemotherapeutic agent is commonly used in combination treatment regimens in different cancer types, of which breast cancer is a prime example [20]. Doxorubicin diffuses passively across the cell membrane, intercalates the DNA and induces cell death. However, since doxorubicin is a weak basic compound the reduced extracellular pH in hypoxic areas of tumors prevents passive drug uptake, referred to as the ion trapping model. This model predicts pH modulating therapies are capable of increasing the uptake and efficacy of doxorubicin [21–23]. Inhibiting CAIX function reduces the degree of extracellular acidification [9, 15, 16, 19, 24], which may thereby increase the uptake and efficacy of doxorubicin. In line with the ion trapping model non-specific carbonic anhydrase inhibition with acetazolamide has been shown previously to increase doxorubicin uptake *in vitro* [18], although the carbonic anhydrase isoform responsible for this effect remained elusive, but was suggested to be CAIX. Furthermore, evidence exist that CAIX inhibition is able to increase doxorubicin efficacy *in vivo* [19].

The ureido-substituted sulfamate S4 is a member of an alternative class of CAIX inhibitory molecules that were synthesized with high selectivity for CAIX [25] and exhibited significant anti-proliferative efficacy *in vitro* in different breast cancer tumor models [26, 27]. Although S4 was ineffective in reducing primary tumor growth *in vivo*, the compound decreased spontaneous lung metastases formation in an orthotopic MDA-MB-231 breast cancer model [25]. This study aims to determine for the first time whether the specific inhibition of CAIX with the sulfamate S4 is able to increase doxorubicin efficacy, both *in vitro* and *in vivo*, and determine if specific CAIX targeting or CAIX knockdown will be able to increase doxorubicin efficacy.

## Methods

### Cell culture

MDA-MB-231 triple negative breast adenocarcinoma (HTB-26), FaDu pharynx squamous cell carcinoma (HTB-43), and HT29 colorectal adenocarcinoma (HTB-38) tumor cells, all obtained from ATCC, were cultured in Dulbecco’s Modified Eagle Medium (DMEM, Lonza) supplemented with 10% Fetal Bovine Serum (FBS, Lonza). Cells were maintained in humidified incubators with 5% carbon dioxide in which they were allowed to attach overnight before the start of experiments.

### Transfection

Inducible genetic *CA9* knockdown (KD) cells were constructed using the pTRIPZ doxycycline inducible system (Open Biosystems). Specific shRNA targeting *CA9*, based on Sigma TRCN0000180210, was inserted in the EcoR1 –Xho1 site of the vector. Lentivirus was made in HEK293FT cells (Thermo Fisher Scientific, # R700-07) and used to infect HT29 cells. These HT29 cells were exposed to low concentrations of doxycycline (1 µg/ml, Sigma-Aldrich) one week prior to the start of experiments to induce a sufficient CAIX KD, as CAIX half-life is approximately 38 hours [28]. HT29 cells exposed to doxycycline, i.e. with a CAIX KD, are defined as HT29 –CAIX low cells, whereas HT29 cells not exposed to doxycycline, i.e. with normal CAIX levels, are defined as HT29 –CAIX high cells.

### Immunoblotting

Adherent cells were exposed to hypoxic conditions for 24 hours in a hypoxic chamber (MACS VA500 microaerophilic workstation, Don Whitley Scientific, UK). The atmosphere in the chamber consisted of 0.2% O_2_, 5% CO_2_ and residual N_2_. Normoxic dishes were incubated in parallel in ambient air with 5% CO_2_. Protein isolates were prepared by scraping cells in RIPA, sonicating the samples, and centrifugation them to remove cellular debris. Protein concentrations were measured using Bradford protein quantification reagent (Bio-rad). Western Blot was performed using primary antibodies against CAIX (M75, kindly provided by Professor Silvia Pastorekova, Institute of Virology, Slovak Academy of Science, Slovak Republic), and beta-actin (MP Biomedicals, #691001) as a reference protein. Primary antibodies were incubated overnight at 4°C, whereas HRP (horseradish peroxidase)-linked secondary antibody (Cell Signalling, #7076) was incubated for 1 hr at room temperature (RT).

### qPCR

Cells were exposed to 0.2% O_2_ for 24 hours after which RNA was isolated using the NucleoSpin^®^ RNA kit (Macherey Nagel). Thereafter cDNA was synthesized using iScript mix (Bio-Rad) and gene expression of CAIX (F-CATCCTAGCCCTGGTTTTTGG, R-GCTCACACCCCCTTTGGTT) and vascular endothelial growth factor (VEGF) (F- GACTCCGGCGGAAGCAT, R- TCCGGGCTCGGTGATTTA) was determined using power SYBR^®^ Green I (Applied Biosystems). Expression of 18S RNA (F- AGTCCCTGCCCTTTGTACACA, R- GATCCGAGGGCCTCACTAAAC) levels was used as a reference.

### Cell viability assays

Tumor cells were seeded with 6000, 1000, or 4000 cells per well for MDA-MB-231, FaDu, or HT29, respectively, in flat bottom 96-well plates. The next day cells were exposed to hypoxia (0.2% O_2_) and medium was replaced by low serum medium (0.2% FBS) containing vehicle (DMSO, final concentration 0.75%), or the sulfamate CAIX inhibitor S4 (33 µM, kindly provided by Professor Claudiu Supuran, University of Florence, Italy). It has previously been demonstrated that 33 µM of S4 was effective in reducing cell viability [25–27]. Simultaneously, cells were exposed to different concentrations of doxorubicin (ranging from 0.01 to 5 µM, Sigma-Aldrich). After 24 hours medium with compounds was replaced with normal DMEM with 10% FBS. Cells were allowed to grow for an additional 72 hrs under normoxic conditions and cell viability was measured thereafter using 3-(4,5-dimethylthiazol-2-yl)-2,5-diphenyltetrazolium bromide (MTT, Sigma-Aldrich) for FaDu, or Alamar Blue (Life Technology) in MDA-MB-231 and HT29 cells. Half the HT29 were exposed to doxycycline (1 µg/ml) one week prior to the experiment and for the entire duration of the experiment to test both HT29 –CAIX high and HT29 –CAIX low cells. Treatment response was quantified in terms of IC50, i.e. the concentration of a drug that gives half-maximal response

### *In vivo* experiments

All animal experiments were ethically approved by the ethical committee on animal experimentation of the university of Manchester (PPL 70/7760) and the university of Maastricht (DEC 2008–025) and performed in accordance to local legislation and guidelines. Eight weeks old NMRI nu/nu mice (Charles River) were subcutaneously injected with MDA-MB-231, FaDu, or HT29 tumor cells resuspended in Matrigel (Corning). Half of the mice implanted with the HT29 cells were provided with water containing doxycycline (2 g/l) and sucrose (5%) ad libitum during the entire experiment to obtain HT29 –CAIX high and HT29 –CAIX low tumor xenografts. For each treatment group 6–8 animals were used. Tumors were measured three times a week in three orthogonal dimensions to calculate tumor volume based on the formula of an ellipsoid. Treatment started when tumors reached a volume of 100–150 mm^3^. A treatment cycle consisted of a ‘five days on, two days off’ schedule with either vehicle (4% DMSO in saline) or S4 (10 mg/kg ip for MDA-MB-231 and FaDu, and 25 mg/kg ip for HT29). Doxorubicin or vehicle control (PBS) was administered ip once a week (5 mg/kg on day 4 of treatment cycle). Total body weight was measured repeatedly for the duration of the study to monitor possible treatment-induced toxicity. In the experiment with the HT29 –CAIX high and HT29 –CAIX low models a parallel group of mice was sacrificed directly after the first treatment cycle, i.e. after the last S4 injection, to investigate levels of CAIX expression in the tumors. Treatment response was quantified as time to reach 2, 5 or 7 times start volume (T2xSV, T5xSV, and T7xSV, respectively) depending on the growth of the tumor model.

### Immunohistochemical staining

Frozen 7 µm tumor sections were fixed in acetone (4°C, 10 min), air-dried and rehydrated in PBS. The sections were incubated with a mixture of polyclonal rabbit anti-CAIX (Novus Biologicals NB100-417) and FITC-conjugated IgG1 mouse monoclonal anti-pimonidazole (clone 4.3.11.3, Hypoxyprobe^™^-1 Plus Kit) antibodies overnight at 4°C, followed by incubation with secondary goat anti-rabbit AlexaFluor 594 (Invitrogen) antibody for 1 hr at RT. Images were acquired as described previously in detail [29].

### Statistical analysis

All statistical analyses were performed using GraphPad Prism (version 5.03). Curve fits of cell viability assays were compared with the extra sum-of–squares F test. Average tumor growth curves were fitted with linear regression. Differences in means between groups were compared with unpaired t-tests. P<0.05 indicates statistical significant difference.

## Results

**Fig 1:**
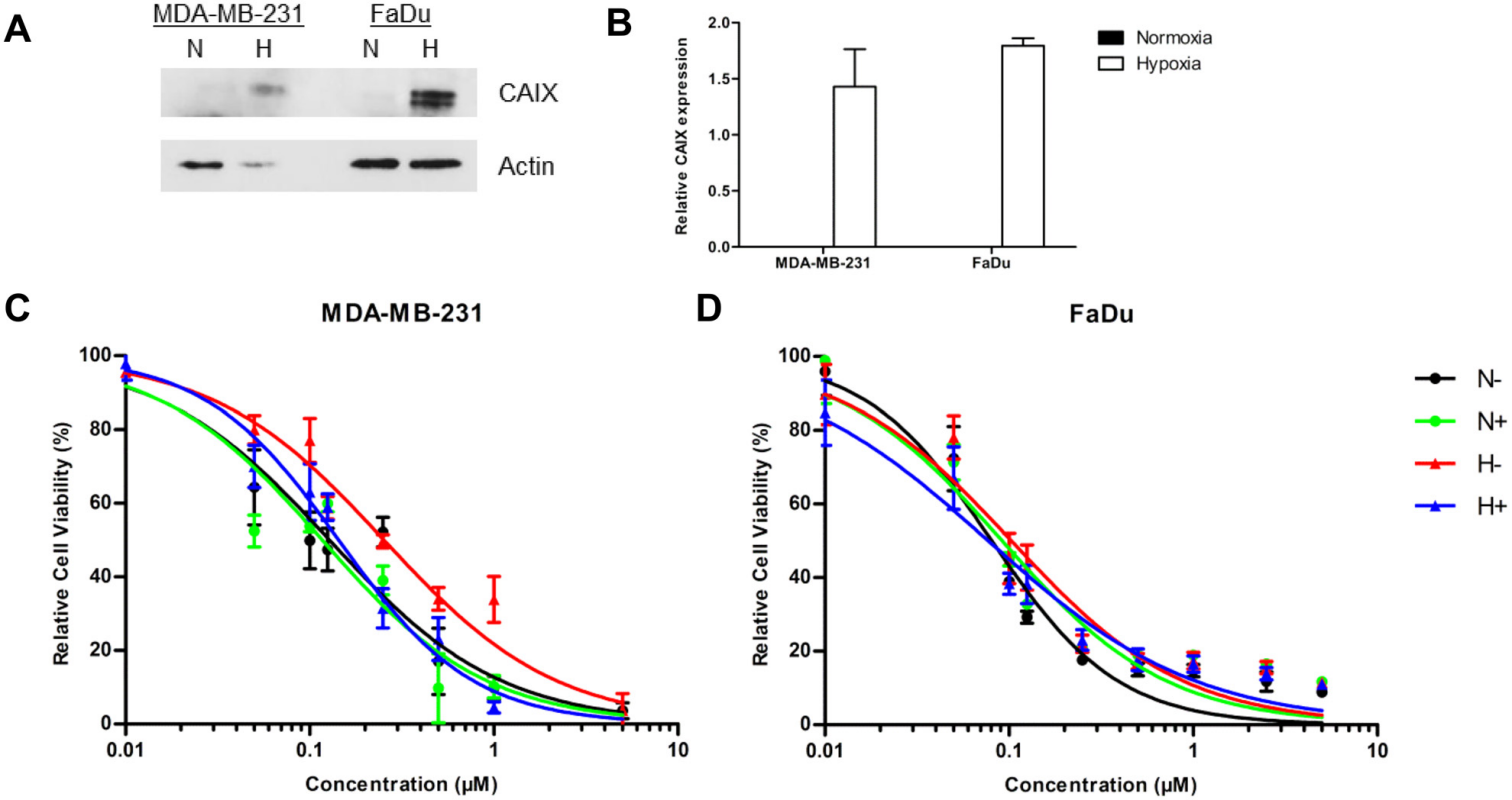
Effect of S4 on doxorubicin sensitivity in MDA-MB-231 and FaDu cells. CAIX protein expression is higher during hypoxia in MDA-MB-231 and FaDu cells (**A**). Quantification of three independent biological repeats shows an almost twofold increase in CAIX expression (normalized to actin expression levels) in both cell lines (**B**). Cell viability assays of MDA-MB-231 (**C**) and FaDu cells (**D**) with increasing concentrations of doxorubicin. Cells were exposed to vehicle (black) or S4 (green) during normoxia (N), or to vehicle (red) or S4 (blue) during hypoxia (H). Results of three independent biological repeats are shown (mean ± SEM).

For both MDA-MB-231 and FaDu cell lines an increased CAIX expression upon hypoxic exposure was observed (Fig 1A and 1B). Cell viability of MDA-MB-231 cells decreased with increasing concentrations of doxorubicin under both normoxic and hypoxic conditions but this effect was slightly more pronounced during normoxia: IC50 0.13 vs. 0.25 µM, p = 0.0025 (Fig 1C). While S4 did not increase the sensitivity of MDA-MB-231 cells during normoxia, IC50 reduced to 0.14 µM during hypoxia (p = 0.0003), suggesting that S4 mediated CAIX inhibition increases doxorubicin efficacy during hypoxia exposure in MDA-MB-231 cells. In contrast, viability of FaDu cells decreased with increasing doxorubicin concentration independent of oxygen concentrations and this effect could not be enhanced by S4 under hypoxia (Fig 1D).

**Fig 2:**
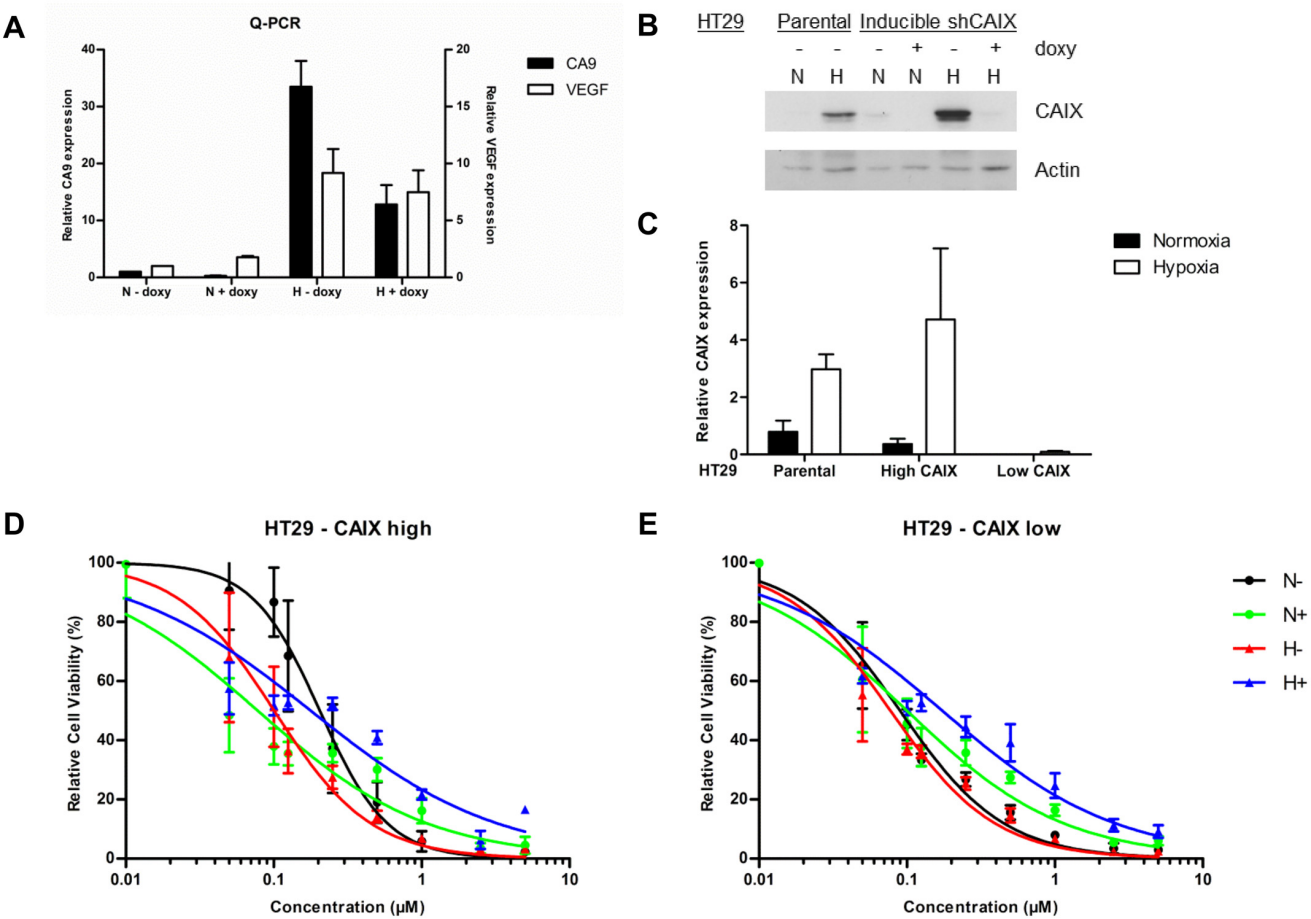
Effect of S4 on doxorubicin efficacy in HT29 –CAIX high and HT29 –CAIX low cells. Exposing HT29 cells to doxycycline (doxy) reduced CA9 mRNA levels during normoxia (N) and hypoxia (H), whereas VEGF levels were unaffected (**A**). CAIX protein levels minimized in HT29 –CAIX low cells when exposed to doxycycline (**B**). Quantification of three independent biological repeats showed minimal residual CAIX protein expression in HT29 –CAIX low cells as compared to parental cells or HT29 –CAIX high cells without a KD (**C**). Cell viability assays of HT29 –CAIX high (**D**) or HT29 –CAIX low cells (**E**) with increasing concentrations of doxorubicin. Cells were exposed to vehicle (black) or S4 (green) during normoxia (N), or to vehicle (red) or S4 (blue) during hypoxia (H). Results of three independent biological repeats are shown (mean ± SEM).

To test if the efficacy of S4 is dependent on CAIX expression, HT29 cells with a doxycycline-inducible knockdown (KD) of *CA9* were generated. HT29 cells were chosen since these cells are shown to be sensitive to CAIX inhibition [15, 16]. Exposing these HT29 cells to doxycycline (1 µg/ml) for one week significantly reduced the mRNA levels of CAIX, but not of a different HIF1-α target, i.e. VEGF, thereby indicating the change in CAIX mRNA levels to be independent on changes in HIF1-α (Fig 2A). Similar results were obtained for protein expression of CAIX which was decreased by doxycycline exposure of HT29 cells both in normoxia and hypoxia (Fig 2B and 2C). Without doxycycline CAIX expression of HT29 cells was however similar to the parental HT29 cells (Fig 2B and 2C). These HT29 cells are therefore defined as HT29 –CAIX high, when not exposed to doxycycline, and HT29 –CAIX low, when a doxycycline-induced CAIX knockdown is present.

Contrary to what was expected, HT29 –CAIX high cells were more sensitive to doxorubicin under hypoxic as compared to normoxic conditions (IC50 0.10 vs 0.20 µM, p = 0.0013) (Fig 2D). Doxorubicin efficacy however did increase when cells were exposed to S4 during normoxic conditions (IC50 0.20 vs 0.08 µM, p<0.0001, Fig 2D), but decreased slightly during hypoxia (IC50 is 0.10 vs 0.18 µM, p = 0.0039, Fig 2D). Sensitivity to doxorubicin under normoxic conditions was increased for HT29 –CAIX low as compared to HT29 –CAIX high cells (IC50 0.09 vs 0.20 µM, p<0.0001, Fig 2D and 2E), suggestive of a CAIX dependent mechanism of increased doxorubicin efficacy. The decreased CAIX expression in HT29 –CAIX low cells might also explain the lack of effect of S4 on doxorubicin sensitivity in these cells during normoxic conditions (IC50 0.09 vs 0.10 µM, p = 0.0972, Fig 2E), or the similar sensitivity during hypoxia (IC50 0.09 vs 0.08 µM, p = 0.6465, Fig 2E). In contrast however, doxorubicin sensitivity decreased when HT29 –CAIX low cells were exposed to S4 during hypoxic conditions (IC50 0.08 vs 0.17 µM, p = 0.0002, Fig 2E). Higher serum concentrations abrogated the effect of S4 on doxorubicin efficacy (S1 Fig), which may be because of the high binding affinity of S4 to bovine serum albumin (data not shown).

**Fig 3:**
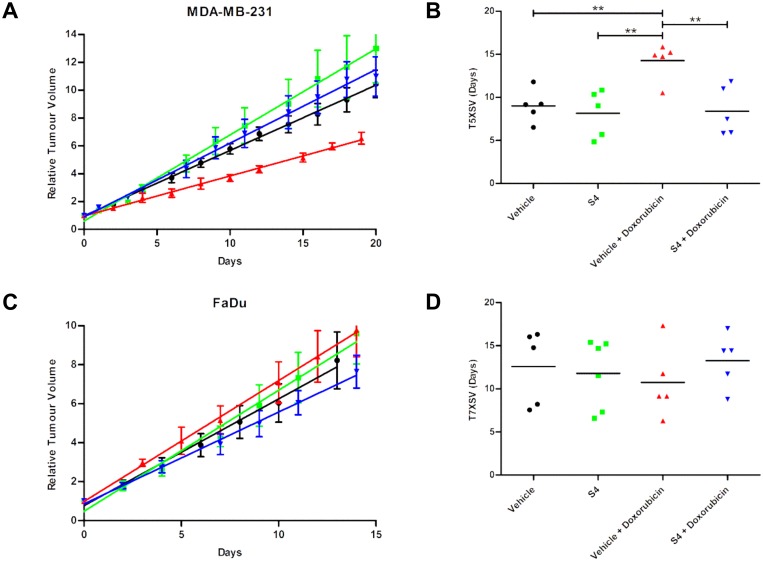
Effect of S4, doxorubicin, or the combination of both, on MDA-MB-231 and FaDu tumor xenograft growth. Relative tumor volume (mean ± SEM) of MDA-MB-231 (**A**) and FaDu (**C**) xenografts treated with vehicle (black), S4 (green), vehicle with doxorubicin (red), or S4 with doxorubicin (blue). Linear fits from relative tumor growth were used to estimate the mean time to reach 5 times start volume (T5XSV) of MDA-MB-231 (**B**) or 7 times start volume (T7XSV) of FaDu (**D**) xenografts. ** indicates statistical significance with p<0.01.

To further study and validate the *in vitro* findings, the combination treatment of S4 with doxorubicin was tested in tumor-bearing mice. No acute toxicity in these animals was observed up to three treatment cycles, as no significant changes in total body weight were observed (S2 Fig). Doxorubicin treatment significantly inhibited MDA-MB-231 tumor growth as compared to vehicle (Fig 3A): time to reach 5 times starting volume (T5xSV) were 14.3 and 8.2 days, respectively (p<0.01, Fig 3B). Contradictory to the *in vitro* data and our hypothesis, S4 treatment abrogated the effect of doxorubicin (p<0.01) in MDA-MB-231. In FaDu xenografts doxorubicin treatment either alone or in combination with S4 was ineffective in reducing tumor growth as compared to vehicle treated mice (T7xSV 10.8, 12.6 and 11.8 days, respectively, Fig 3C and 3D).

**Fig 4:**
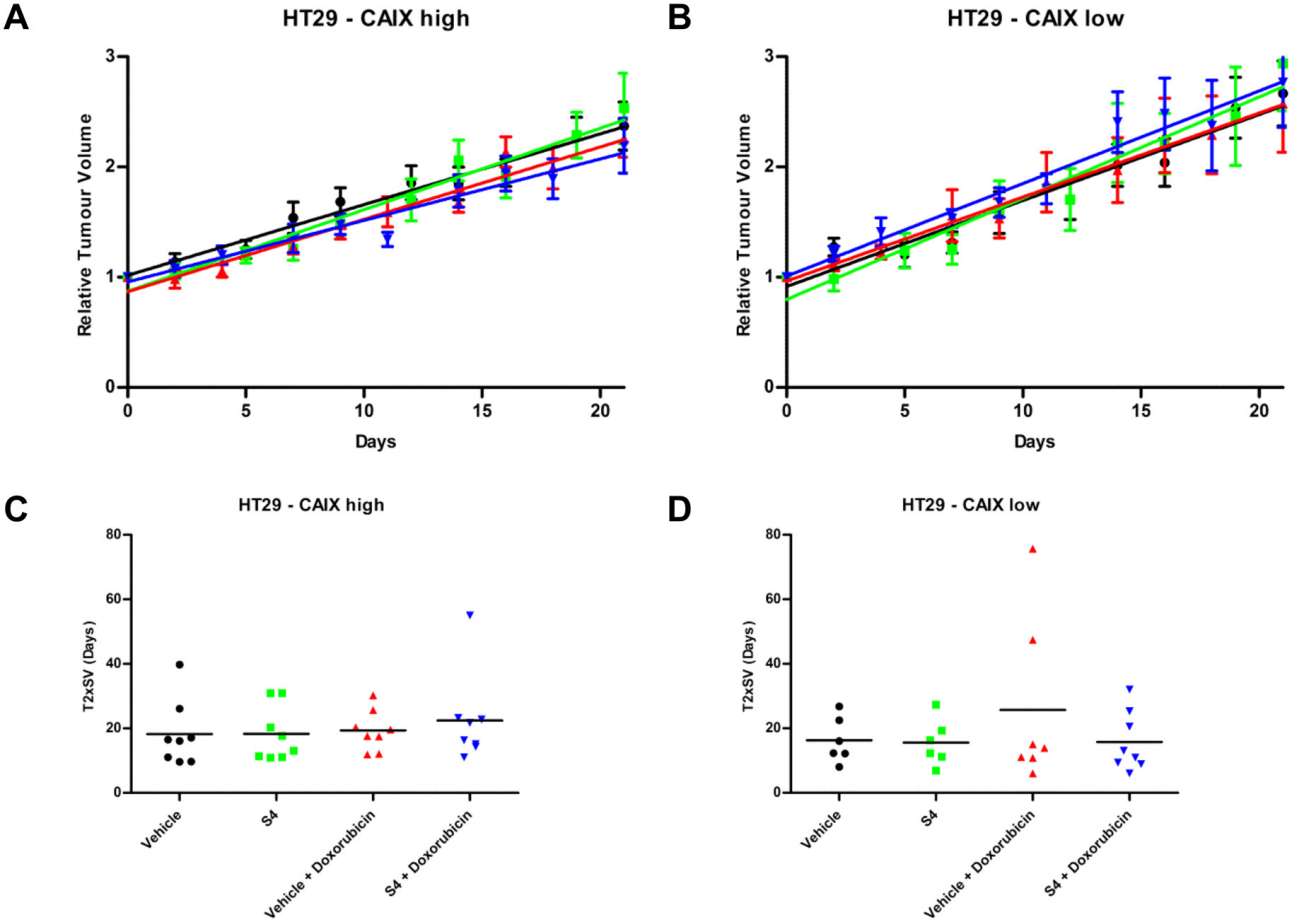
Effect of S4, doxorubicin, or the combination of both, on HT29 –CAIX high and HT29 –CAIX low tumor xenograft growth. Relative tumor volume (mean ± SEM) of HT29 –CAIX high (**A**) or HT29 –CAIX low xenografts (**B**) treated with vehicle (black), S4 (green), vehicle with doxorubicin (red), or S4 with doxorubicin (blue). Linear fits from relative tumor growth were used to estimate the mean time to reach 2 times start volume (T2XSV) of HT29 –CAIX high (**C**) or HT29 –CAIX low (**D**) xenografts.

To test whether the efficacy of combined treatment of doxorubicin with S4 *in vivo* depends on CAIX expression we employed the HT29 –CAIX high and HT29 –CAIX low tumor models. The HT29 –CAIX low tumor model exhibited a markedly reduced expression of CAIX by doxycycline exposure in the drinking water, although residual CAIX expression could still be observed. In both the HT29 –CAIX high and HT29 –CAIX low tumor models pimonidazole-positive labeling demonstrated presence of hypoxia (S3 Fig). Single S4 or doxorubicin treatment did not inhibit the growth of HT29 –CAIX high (Fig 4A) or HT29 –CAIX low (Fig 4B) tumors. Tumor response to doxorubicin treatment was not improved by knockdown of CAIX as time to reach 2 times start volume (T2XSV) was not statistically different (19.4 vs 25.7 days, p = 0.5144, Fig 4C and 4D). In addition, combination of S4 with doxorubicin was unable to increase doxorubicin efficacy in HT29 –CAIX high (T2XSV 19.4 vs 22.4, p = 0.5801, Fig 4C), or in HT29 –CAIX low tumors (T2XSV 25.7 vs 15.8, p = 0.3274, Fig 4D). Additional treatment cycles were also ineffective in exerting an effect on tumor growth and caused severe toxicity in animals.

## Discussion

In the present study we investigated whether doxorubicin treatment efficacy can be enhanced when combined with the CAIX specific sulfamate inhibitor S4 [25–27, 30]. Previously, CAIX inhibition has been shown to potentiate the efficacy of standard treatment modalities, such as radio- and chemotherapy [15, 16, 19]. Furthermore, non-isoform specific CA inhibition with acetazolamide can increase doxorubicin uptake, thereby making it more effective [18]. The ion trapping model explains the higher efficacy of the weak basic anthracycline antitumor antibiotic doxorubicin due to an increased uptake when the balance between intracellular and extracellular pH is shifted towards a reduced extracellular acidification upon hypoxia when treated with selective CAIX inhibitors [21–23]. The results of this study demonstrate that the selective CAIX inhibitor S4 increased doxorubicin efficacy *in vitro* in MDA-MB-231 cells, specifically in hypoxic conditions. These results could however not be found in FaDu, HT29 –CAIX low and HT29 –CAIX high cells; although for the latter an increased efficacy of doxorubicin was observed during normoxia when combined with S4. This increase in doxorubicin efficacy therefore appeared to depend on the levels of CAIX expression in normoxic HT29 cells. Doxorubicin efficacy however was not modulated by S4 under hypoxic conditions, which are essential for full CAIX activation [24, 31]. Nevertheless, this could be due to an incomplete CAIX inhibition that would still reduce the extracellular pH and thereby reduce doxorubicin uptake and efficacy.

To validate the efficacy of S4 treatment *in vivo* experiments were conducted using the same cell lines, since tumor hypoxic and acidic microenvironment might be an important factor influencing the potential of S4 to enhance doxorubicin cytotoxicity. Similar to the previous report [25] S4 monotherapy did not inhibit the growth of any of the tumor models included in this study. These results are in line with histological evaluation of tumor microenvironmental characteristics in SCCNij202 xenografts demonstrating that S4 treatment did not have an effect on proliferation, apoptosis, necrosis, and hypoxia [32]. Taken together the data indicate that S4 lacks anti-cancer efficacy *in vivo* in primary tumors. Previously S4 has been described to reduce migration of MDA-MB-231 tumor cells, and prevent invasion of *ex vivo* breast cancer spheroids [25, 27]. Furthermore, treatment of orthotopic MDA-MB-231 xenografts with S4 reduced the number of spontaneous lung metastases [25]. In addition, it has been shown that S4 can increase ectodomain shedding of CAIX, both *in vitro* and *in vivo*, although the exact relevance and mechanism of this finding requires additional studies [30, 32].

In this study doxorubicin treatment was only effective in reducing tumor growth in the MDA-MB-231 xenografts, whereas no effect was observed in the FaDu, HT29 –CAIX high and HT29 –CAIX low tumor models. This difference between models is unlikely caused by any intrinsic variation in doxorubicin sensitivity, as no differences were observed *in vitro* or in previous reports [33]. The tumor microenvironment might therefore be responsible for this difference in doxorubicin response. Quantification of the exogenous hypoxia marker pimonidazole in FaDu xenografts revealed the average hypoxic fraction to range between 10–20% [34–36]. The hypoxic fraction of the subcutaneous HT29 –CAIX high tumors was also approximately 10% and increased to 20% in the HT29 –CAIX low tumors in previous experiments (data not shown). In contrast however, orthotopic MDA-MB-231 xenografts implanted in the mammary fat pad have a hypoxic fraction of only 5% [37], whereas the hypoxic fraction is estimated to be approximately 7% for subcutaneous tumors (data not shown). Hypoxic areas in tumors are characterized by extracellular acidification, which would reduce doxorubicin uptake and thereby decrease its efficacy. The relatively lower amount of hypoxia in MDA-MB-231 tumors as compared to FaDu and HT29 tumors could therefore explain the reduced efficacy of doxorubicin in the latter tumor model. Furthermore, the difference in doxorubicin efficacy between HT29 and MDA-MB-231 tumors can likewise be explained by a relatively higher activity of CAIX in HT29 cells as compared to the MDA-MB-231 cells [18], leading to a higher degree of extracellular acidification and as a result reduced doxorubicin efficacy. This is further supported by the results obtained in spheroid models showing that MDA-MB-231 cells are more sensitive to doxorubicin as compared to HT29 cells [38].

Treating HT29 tumor-bearing mice with doxorubicin was reported previously to be effective [19]. The lack of effect observed in this study might partly be explained by retention of doxorubicin in the abdominal cavity after intraperitoneal injections [39], as opposed to intravenous injections used previously [19]. This alternative administration route might therefore prevent adequate distribution of doxorubicin to xenograft tumors with poorer vasculature and higher hypoxic fraction, i.e. FaDu and HT29. We were unable to increase the doses of doxorubicin in this study due to severe cardiac toxicity caused by doxorubicin after multiple treatment cycles [40].

In conclusion, treatment with the sulfamate CAIX inhibitor S4 has no monotherapeutic effect and is unable to increase doxorubicin efficacy. In contrast, S4 abrogated the effect of doxorubicin in MDA-MB-231 tumors. The mechanism underlying this reduction in doxorubicin efficacy remains unclear. Nevertheless, these results, in combination with the pharmacokinetic characteristics of S4 after oral and intravenous administration (S4 Fig) suggest sufficient concentrations of S4 might have reached the tumor. Previously a different class of CAIX inhibitor has been shown to increase doxorubicin efficacy and also exert an effect as single agent therapy [19]. These contradictory findings stress the importance of further investigations to better understand the mechanism of action of different classes of CAIX inhibitors before being implemented as anti-cancer agents in combination with standard treatment modalities such as doxorubicin treatment.

## Supporting Information

S1 Fig Cell viability assay of the HT29 –CAIX high and HT29 –CAIX low cells with 10% FBS. Cell viability assays of HT29 –CAIX high (**A**) or HT29 –CAIX low cells (**B**) with increasing concentrations of doxorubicin. Cells were exposed to vehicle (black) or S4 (green) during normoxia (N), or to vehicle (red) or S4 (blue) during hypoxia (H). Results of three independent biological repeats are shown (mean ± SEM).
(TIF)

S2 Fig Relative total body weight of mice with different tumor xenografts. Mice were implanted with MDA-MB-231 (**A**), FaDu (**B**), or HT29 –CAIX high (**C**) or HT29 –CAIX low (**D**) xenografts. Relative total body weight (mean ± SEM) of mice treated with vehicle (black), S4 (green), vehicle with doxorubicin (red), or S4 with doxorubicin (blue) showed no signs of acute toxicity in any of the treatment groups for any of the included tumor models.
(TIF)

S3 Fig Validation CAIX expression in HT29 –CAIX high and HT29 –CAIX low xenografts. CAIX expression (red) and pimonidazole-labelled hypoxia (green) in the HT29 –CAIX high tumors, and the HT29 –CAIX low xenografts by addition of doxycycline in the drinking water of the mice.
(TIF)

S4 Fig Pharmacokinetic studies with S4. A single dose of S4 was administered either intravenously (5 mg/kg) (**A**) or orally (50 mg/kg) (**B**) in male CD1 mice. S4 was dissolved in 12.5% ethanol, 37.5% triethylene glycol, and 50% saline. Blood samples were taken at 7 different time points (n = 3 mice per time point) after injection and plasma was isolated. Plasma was mixed with methanol, centrifuged and the supernatant was transferred to mass spectrometry plate for LC-MS/MS analysis. From the single intravenous administration several parameters could be estimated (**C**). From the oral administration these parameters could not be estimated because the concentration curve over time was too inaccurate. The curve after oral injection however does suggest S4 to be slowly resorbed out of the intestine. Intraperitoneal injection of S4 might therefore form a depot at the injection site, thereby only releasing limited concentrations in the blood and eventually reaching the tumor. S4 might thereby only exert an effect in certain sensitive tumor models. These studies were performed by Cyprotex Ltd. (Macclesfield, UK) as a part of the EU 7th framework program METOXIA (ref. 2008–222741) funded initiative.
(TIF)

S1 File Raw data from cell viability assays doxorubicin with or without S4. The data of all three independent biological repeats is included of all cell lines described in the manuscript.
(XLSX)

S2 File Raw data of CAIX and VEGF gene expression of the inducible genetic *CA9* knockdown HT29 cells. Q-PCR data of three independent repeats is included of HT29 –CAIX high and HT29 –CAIX low cells exposed to normoxic and hypoxic conditions.
(XLSX)

S3 File Measured tumor volume and total body weight of MDA-MB231 tumor-bearing mice. Tumor volumes were normalized to start of treatment and tumor growth was fitted with linear regression fits to extrapolate the time to reach 5 times start volume (T5xSV). Group 1 was treated with vehicle, group 2 was treated with S4, group 3 was treated with doxorubicin in combination with vehicle, and group 4 was treated with the combination of doxorubicin with S4.
(XLS)

S4 File Measured tumor volume and total body weight of FaDu tumor-bearing mice. Tumor volumes were normalized to start of treatment and tumor growth was fitted with linear regression fits to extrapolate the time to reach 7 times start volume (T7xSV). Group 1 was treated with vehicle, group 2 was treated with S4, group 3 was treated with doxorubicin in combination with vehicle, and group 4 was treated with the combination of doxorubicin with S4.
(XLS)

S5 File Measured tumor volume and total body weight of HT29 –CAIX high and HT29 –CAIX low tumor-bearing mice. Tumor volumes were normalized to start of treatment and tumor growth was fitted with linear regression fits to extrapolate time to reach 2 times start volume (T2xSV). Doxycycline (Doxy) was administered in the drinking water to induce a CAIX knockdown. Doxorubicin is abbreviated as Doxo, where +/- indicates compound or vehicle treatment, respectively.
(XLSX)

S6 File Raw data of pharmacokinetic analyses of S4 administration. Concentration of S4 were measured at the indicated time points (n = 3 mice per time point) after both intravenous and oral administration of S4.
(XLSX)
