# Supplementary figures and images for: The Sulfamate Small Molecule CAIX Inhibitor S4 Modulates Doxorubicin Efficacy
Source: PLoS One. 2016 Aug 11;11(8):e0161040. doi: 10.1371/journal.pone.0161040 (PMC4981362; doi:10.1371/journal.pone.0161040)

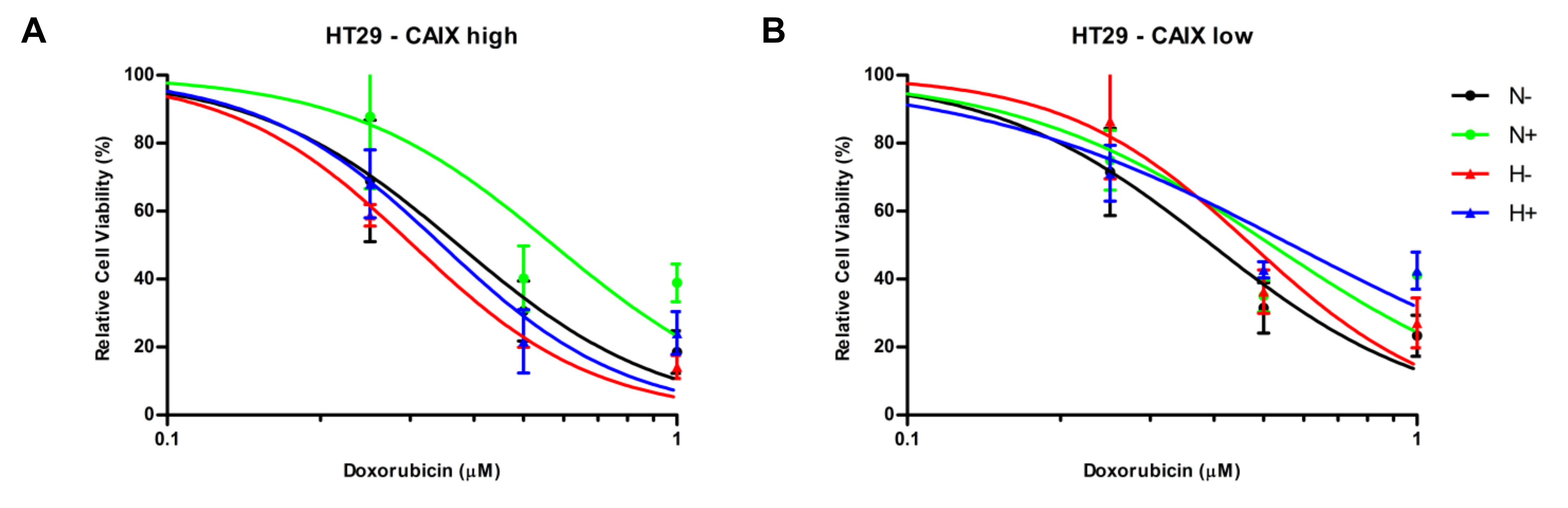

Supplement: S1 Fig — Cell viability assays of HT29 –CAIX high (A) or HT29 –CAIX low cells (B) with increasing concentrations of doxorubicin. Cells were exposed to vehicle (black) or S4 (green) during normoxia (N), or to vehicle (red) or S4 (blue) during hypoxia (H). Results of three independent biological repeats are shown (mean ± SEM). (TIF) [file pone.0161040.s001.tif]

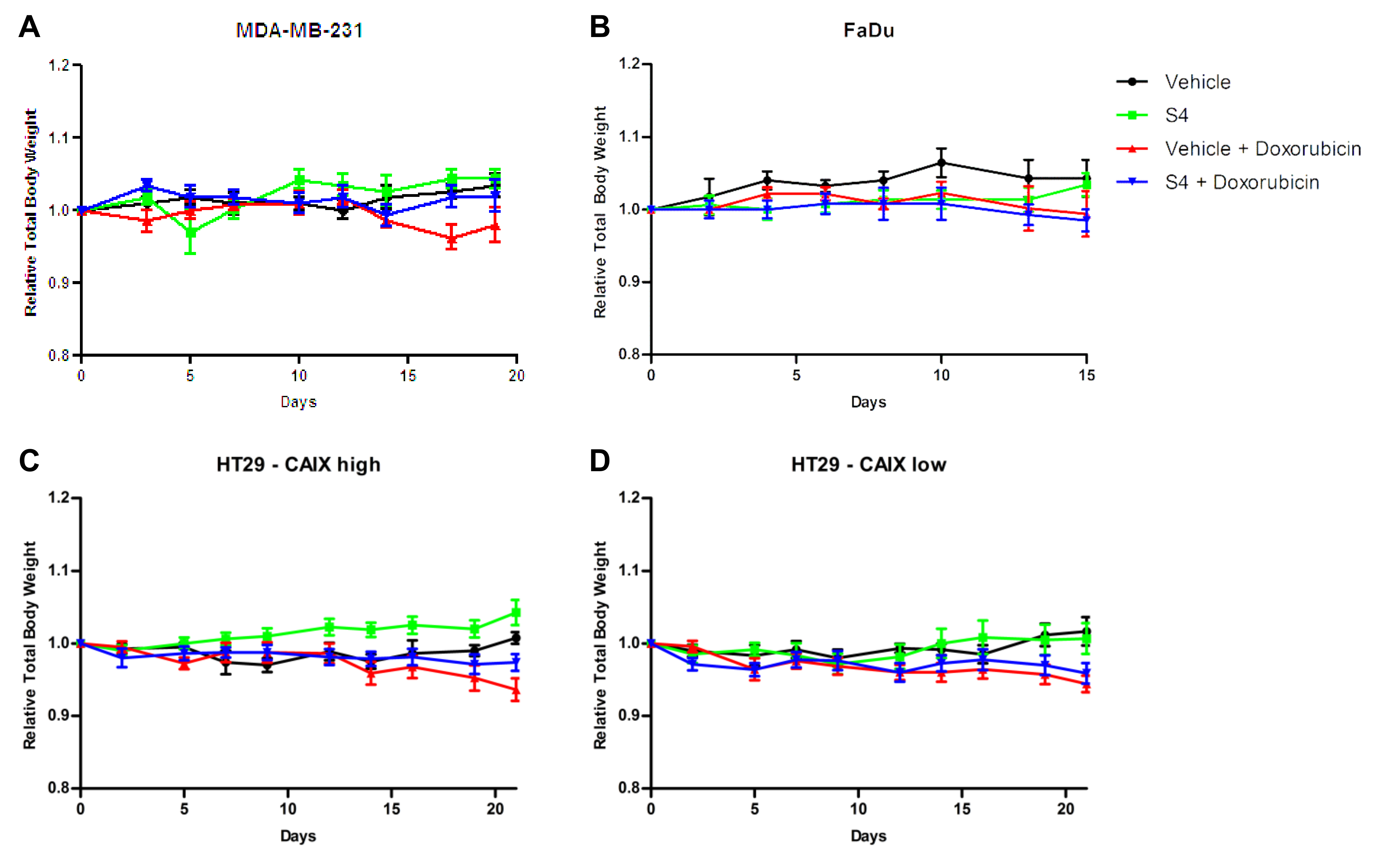

Supplement: S2 Fig — Mice were implanted with MDA-MB-231 (A), FaDu (B), or HT29 –CAIX high (C) or HT29 –CAIX low (D) xenografts. Relative total body weight (mean ± SEM) of mice treated with vehicle (black), S4 (green), vehicle with doxorubicin (red), or S4 with doxorubicin (blue) showed no signs of acute toxicity in any of the treatment groups for any of the included tumor models. (TIF) [file pone.0161040.s002.tif]

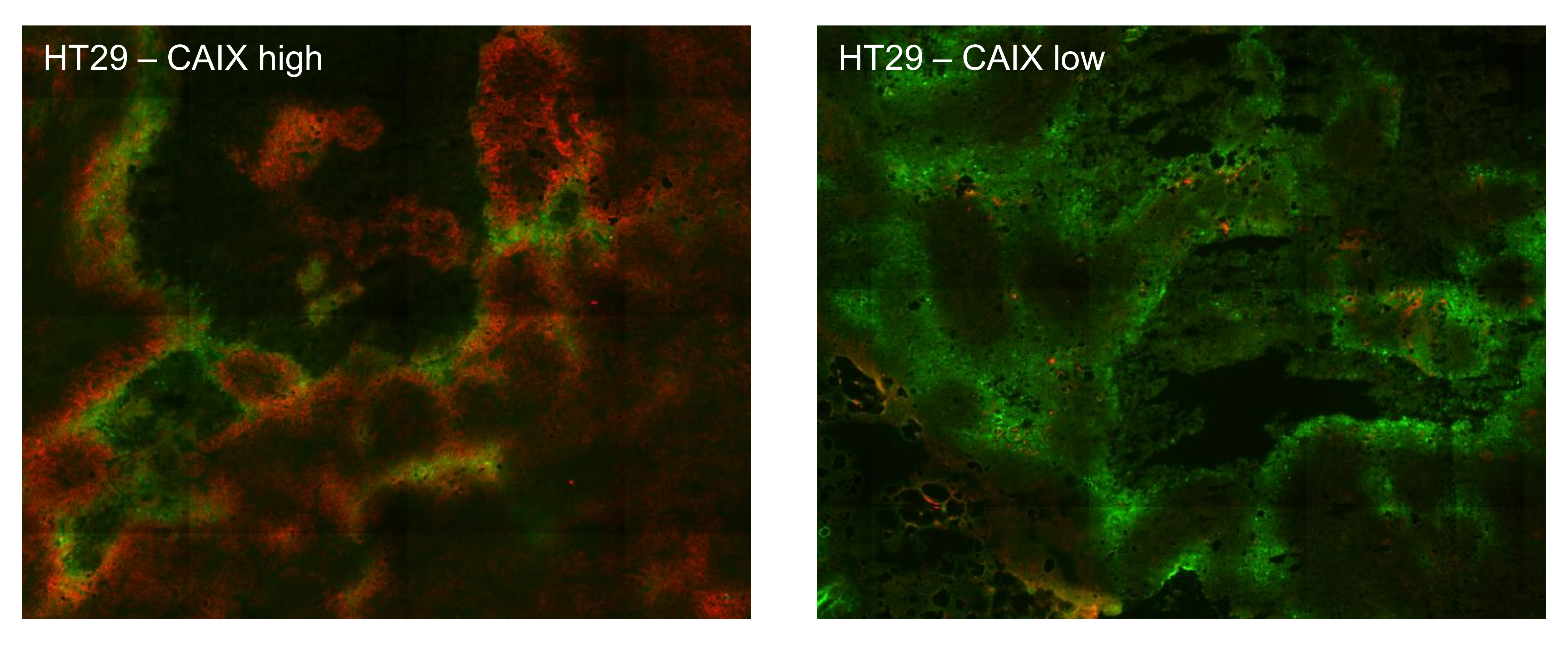

Supplement: S3 Fig — CAIX expression (red) and pimonidazole-labelled hypoxia (green) in the HT29 –CAIX high tumors, and the HT29 –CAIX low xenografts by addition of doxycycline in the drinking water of the mice. (TIF) [file pone.0161040.s003.tif]

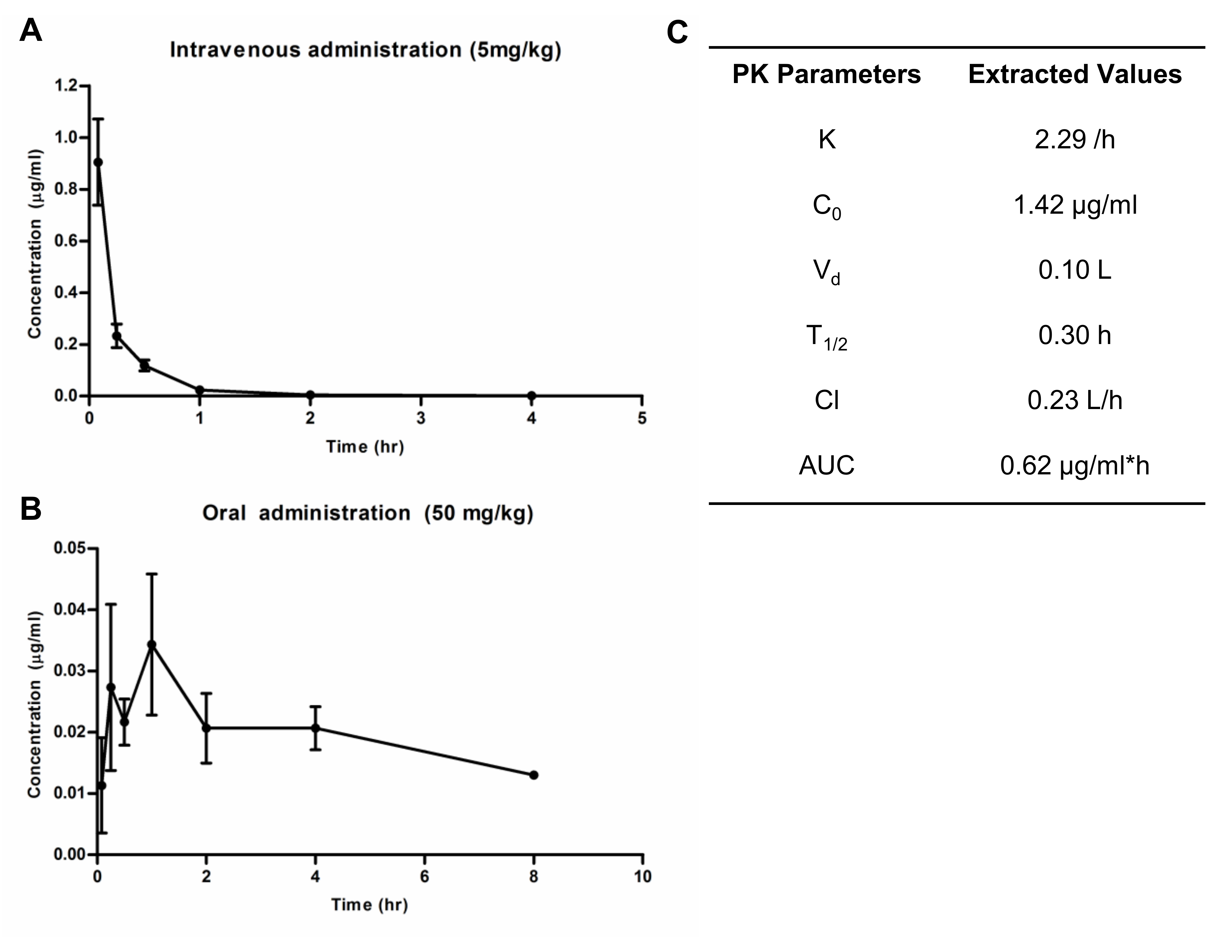

Supplement: S4 Fig — A single dose of S4 was administered either intravenously (5 mg/kg) (A) or orally (50 mg/kg) (B) in male CD1 mice. S4 was dissolved in 12.5% ethanol, 37.5% triethylene glycol, and 50% saline. Blood samples were taken at 7 different time points (n = 3 mice per time point) after injection and plasma was isolated. Plasma was mixed with methanol, centrifuged and the supernatant was transferred to mass spectrometry plate for LC-MS/MS analysis. From the single intravenous administration several parameters could be estimated (C). From the oral administration these parameters could not be estimated because the concentration curve over time was too inaccurate. The curve after oral injection however does suggest S4 to be slowly resorbed out of the intestine. Intraperitoneal injection of S4 might therefore form a depot at the injection site, thereby only releasing limited concentrations in the blood and eventually reaching the tumor. S4 might thereby only exert an effect in certain sensitive tumor models. These studies were performed by Cyprotex Ltd. (Macclesfield, UK) as a part of the EU 7th framework program METOXIA (ref. 2008–222741) funded initiative. (TIF) [file pone.0161040.s004.tif]
